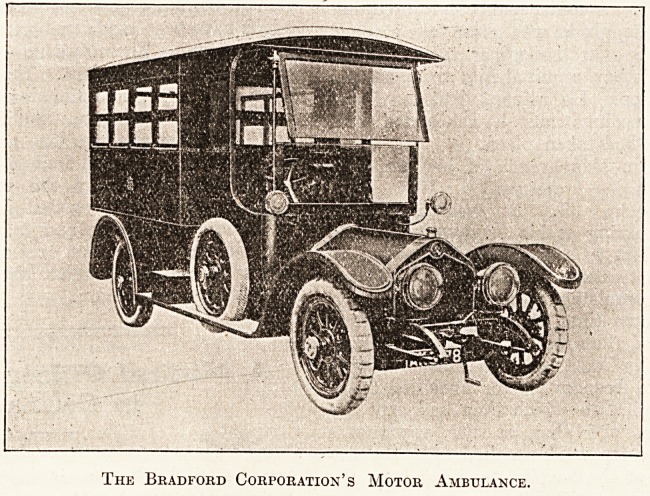# Practical Points in Hospital Matters: An Improved Type of Motor Ambulance

**Published:** 1915-01-16

**Authors:** 


### PRACTICAL POINTS IN HOSPITAL MATTERS.

(Criticism and Suggestions Invited.)

### An Improved Type of Motor Ambulance

The effect of employing motor ambulances and electric motor carriers for the service of patients' food, which have been illustrated and described in previous issues of The Hospital, has been to improve the construction of the appliances in nearly every respect. It is, therefore, interesting to study the recent type of motor ambulance which the City Corporation of Bradford has installed for general municipal work. It exhibits many improvements which cannot fail to interest those engaged in ambulance work.

In the first place this ambulance has a convertible interior, which can be quickly changed from a comfortable carriage with cushioned seats and back-rests for crippled children into an ambulance proper with a sterilisable interior—composed of a zinc lining which is finished in hard white enamel —with steel stretchers and air-beds that are provided with waterproof covers.

The Bradford Corporation's Motor Ambulance.

### Its Advantages for Municipal Work.

This car comprises a 25-h.p. Crossley chassis, complete with steel splash wings and vallances, spare wheel and bracket, and is fitted with 895 by 135 Dunlop tyres (two steel-studded). The exterior is finished in very dark blue. Ventilation is secured by means of patent regulators to the windows, and the interior gear comprises two steel concave stretchers with tubular carrying handles. All corners inside the car are filleted 1-in. radius to facilitate easy cleansing, and each stretcher is mounted on a patent swing gear with extension, enabling the stretcher to be drawn centrally to the door opening and loaded from the outside of the car, thus obviating any climbing up into the car on the part of the forward bearer. The equipment also includes a metal-enamelled detachable seat for a nurse or attendant, and when the stretchers are removed the seats are fitted with loose spring and with hair-stuffed cushions.

It must be remembered in this connection that municipal ambulance work is varied, and has to provide for a number of contingencies that, in the old days, one vehicle would not have been expected to perform. Street accidents, conveyance perhaps of cases to isolation hospitals, what is virtually "passenger traffic" between institutions and convalescent homes, the transference of cripples, these are but obvious indications of the diverse work required.

The type of car illustrated can be fitted, it is claimed, to any chassis of moderate horsepower. The work has been carried out by J. and A. Carter, 2-6 New Cavendish Street, London, who have developed their business and usefulness as invalid furniture makers by undertaking the design and construction of these ambulances. Their design has proved practical for the hard wear required in the Colonies, and local authorities as well as hospitals will be interested in this design.

Now that so many public authorities—particularly at places, like Bradford, which are not far from the East Coast—are placing public buildings at the Government's disposal, the ambulances possessed by these authorities are likely to be more severely tested than was thought possible a few months ago. It is another triumph for the motor in warfare that, as is now known, the motor ambulance proves superior also for this work.

## Figures and Tables

**Figure f1:**